# The Influence of Solar Sintering on Copper Heat Exchanger Parts with Controlled 3D-Printed Morphology

**DOI:** 10.3390/ma15093324

**Published:** 2022-05-05

**Authors:** Mihai Alin Pop, Cătălin Croitoru, Tibor Bedo, Virgil Geamăn, Irinel Radomir, Aurel Crișan, Emmanuel Guillot, Ioan Miloșan, Sebastian Marian Zaharia, Lucia Antoaneta Chicoș

**Affiliations:** 1 Materials Science Department, Transilvania University of Brasov, 29 Eroilor Ave., 500484 Brasov, Romania; mihai.pop@unitbv.ro (M.A.P.); bedo.tibor@unitbv.ro (T.B.); geaman.v@unitbv.ro (V.G.); crisan.a@unitbv.ro (A.C.); milosan@unitbv.ro (I.M.); 2 Materials Engineering and Welding Department, Transilvania University of Brasov, 29 Eroilor Ave., 500484 Brasov, Romania; 3 Mathematics and Informatics Department, Transilvania University of Brasov, 29 Eroilor Ave., 500484 Brasov, Romania; i.radomir@unitbv.ro; 4 PROMES-CNRS Laboratory, Processes, Materials and Solar Energy, 7 Rue du Four Solaire, F-66120 Font Romeu Odeillo, France; emmanuel.guillot@promes.cnrs.fr; 5 Manufacturing Engineering Department, Faculty of Technological Engineering and Industrial Management, Transilvania University of Brasov, 29 Eroilor Ave., 500484 Brasov, Romania; zaharia_sebastian@unitbv.ro (S.M.Z.); l.chicos@unitbv.ro (L.A.C.)

**Keywords:** powder metallurgy, 3D printing, concentrated solar energy

## Abstract

From a scientific point of view, heat transfer is different in solar furnaces compared with classical ones and the influence of direct concentrated solar radiation on sintered parts needs to be studied in detail to determine the feasibility of solar furnaces in manufacturing small workpieces. This study was performed on cylindrical samples with controlled morphology obtained by a powder metallurgy 3D printing technique. All samples were heated with a heating rate of 120 ± 10 °C/minute, with 0, 1, 2, 3, 4 and 5 min holding times at 900 °C and 930 °C. The morphology of the samples was analyzed microscopically, the microhardness was determined before and after sintering, and the results were correlated with the sintering parameters (temperature, heating rate and holding time). The best results were obtained at 930 °C with 5 min holding time from the microhardness value and microstructure point of view.

## 1. Introduction

Three-dimensional printing is a unique technology that offers a high degree of freedom for customizing practical products in a short time at an acceptable price. In recent years, the improved and reliable 3D printers and 3D scanners custom-made for additive manufacturing technology are becoming an increasingly viable and cost-effective option for high-mix–low-volume manufacturing of customized parts and prototypes [1,2,3].

These new technologies have been collectively classified as additive manufacturing (AM), which seems to be the most commonly used name, and as rapid prototyping (RP) technologies and solid freeform fabrication (SFF) techniques. These techniques with reproducible and mathematically predictable physical properties have become a fast-developing research area [4,5].

Computer-aided designs are applied to obtain precise geometries, with the possibility to obtain different shapes.

The design is imported into a software, which mathematically slices the conceptual model into horizontal layers. Toolpaths are generated before the data are downloaded to the Filament Fused Fabrication (FFF) hardware. The FFF extrusion head operates in the X and Y axes while the platform is lowered in the Z-axis in order to form each new layer. Practically, the process draws the designed model one layer at a time [1,2,3,4,5].

The focus of the present paper is to investigate how solar energy can be used to sinter copper parts with internal controlled geometry via powder metallurgy. These parts are obtained by using a negative 3D model, which is printed using polyvinyl alcohol (PVA)-based resin filament and filled with different metallic powders. Before sintering, the support material must be removed (PVA) by immersing it in water until it is totally eliminated, and then, the part is dried and sintered in the furnace. By sintering, the resulting part will have a special internal architecture given by the 3D-printed model.

A cellular architecture with deterministic 3D morphology was developed by our research group in recent years for different prototypes to create precise models with well-defined architectures [6,7].

On the other hand, powder metallurgy is also an evolving technology, using a wide variety of metallic and alloy materials [8,9,10].

By producing parts using the PM process, we can obtain a homogeneous structure, with controlled porosity and special properties such as hardness and wear resistance, for a wide range of applications, especially for the automotive industry [11,12,13,14].

The action of concentrating the solar energy on small surfaces offers the possibility of local heating necessary for obtaining these types of metallic parts. Besides obtaining the proposed features for these types of materials, the exploitation of advantages given by using solar energy compared with the classical ones is taken into consideration [15,16]. Environmental protection by reducing the pollution level while exploiting the technical availabilities of using solar energy is also desirable.

The compaction of powders (semi-finished products) subjected to sintering for the models obtained by 3D printing have the advantage that by 3D printing, parts (shapes, molds and models) with any configuration (with complex surfaces) without the need for further machining or raw models can be obtained directly from a computer drawing and in a very short time. This considerably reduces the execution time of the profiled molds used for compacting the powders and, finally, the time for obtaining the sintered parts. Sintering in solar ovens in turn reduces the time; energy consumption; labor; and therefore, the costs of the final parts.

Starting from these premises, we researched how to obtain parts made from Cu (99.6% powder purity), “full” parts (without internal gaps) and parts with thin walls (with internal gaps) by solar sintering.

The concrete objectives of the research were to obtain and establish the working parameters for the sintering in solar ovens with concentrated energy for copper parts with or without internal gaps and the analysis of the factors that influence their structure and properties.

Numerous researches have been found in the literature regarding the classical sintering of Cu or Cu matrix composite materials where the optimal temperature was determined to be 900 °C, coupled with a holding time of 120 min [17]. Additionally, it has been found that the relative density and hardness of sintered parts increase with the sintering temperature [10].

The values of the micro hardness for conventionally furnace-sintered pure Cu are 43 ± 2.6 HV_100_, and by microwave sintering at 900 °C, it is 46 ± 2.8 HV_100_ with a holding time of 60 min [12,18,19,20,21,22].

The main objectives of this paper are to establish technological parameters for sintering Cu metallic powders for the manufacture of prototypes with internal morphology using 3D printing technology under concentrated solar energy (CSE) action in relation to the pre-established properties.

## 2. Experimental

### 2.1. Materials and Equipment

**Figure 1:**
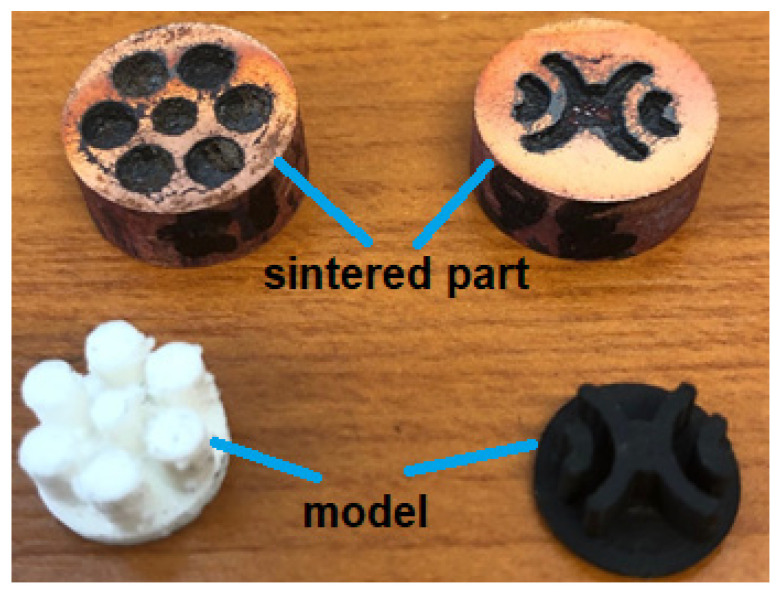
Different internal sample architectures.

The research was carried out on cylindrical specimens (samples) with dimensions D × H = 16 mm × 30 mm (Figure 1). The metal powder is Cu > 99% (purchased from Alfa Aesar, Thermo Fisher Scientific, Shore Road, Port of Heysham Industrial Park Lancashire, LA3 2XY, Heysham, Lancashire, UK) with size of <63 µm [23].

A CreatBot (Henan Creatbot Technology Limited 6#, Chaoya Industry Park, Hanghai Road, No.13 Ave., Eco-Tech Development Zone, Zhengzhou City, Henan Province, China) DX—a 3D double-nozzle printer—was used to obtain models with a 0.2 mm layer thickness and PVA material (Filament AquaSolve™—PVA Natural) from Form Futura Company (Tarweweg 3, 6534 AM Nijmegen, The Netherlands) [24]. The printing temperature for the filament (measured at the extrusion head) was 200 °C and a bed temperature of 50 °C with a printing speed of 40 mm/s, as recommended by the manufacturer.

For pressing, a universal testing machine, a type WDW-150S (Jinan Testing Equipment IE Corporation, Jinan, China) was used, and for drying, a Nabertherm furnace (Nabertherm GmbH, Bahnhofstr. 20, 28865, Lilienthal, Germany) was used.

A Delta Wasp 2040 Clay 3D printer (Via Castelletto, 104, 48024 Massa Lombarda RA, Italy) with a 0.5 mm layer thickness was used to manufacture a ceramic crucible starting from a ceramic slurry, followed by sintering at 1350 °C in the Nabertherm furnace.

Solar sintering was performed at Odeillo-Font Romeu (PROMES-CNRS, 7, rue du Four Solaire, 66120 Font Romeu Odeillo, France) using MSSFs furnace with a diameter of 16 mm of the concentrated solar energy focused beam.

A FM-700 AHOTEC tester (Future-Tech Corp, Talkpier Kawasaki, Kanagawa, Japan) was used to determine the microhardness with 100 gf for 10 s.

### 2.2. Technological Stages of Obtaining the Copper Parts

The copper-sintered parts were obtained following the six-step protocol mentioned below:

1. The CAD model (SolidWorks 2016 software) was designed, and a mold (negative part) was printed by 3D printing using FFF technology (PVA material).

2. The cavity die of this mold (the negative part) was filled gravimetrically with metallic powder.

3. The powder was pressed unidirectional in the die at a force of F = 90 kN. The pressing aimed to obtain a sufficient mechanical strength for the next three steps.

4. The support material (PVA) was removed by immersion in water until complete dissolution for different time periods, depending on the amount, size of the sample and water temperature (roughly 48 h).

5. The drying process was performed in the Nabertherm furnace at 120 °C with a holding time of 2 h.

6. The dried parts were sintered in the CSE furnace and purged with N_2_. The N_2_ flow was 5 L/min to prevent oxidation of the samples.

**Figure 2:**
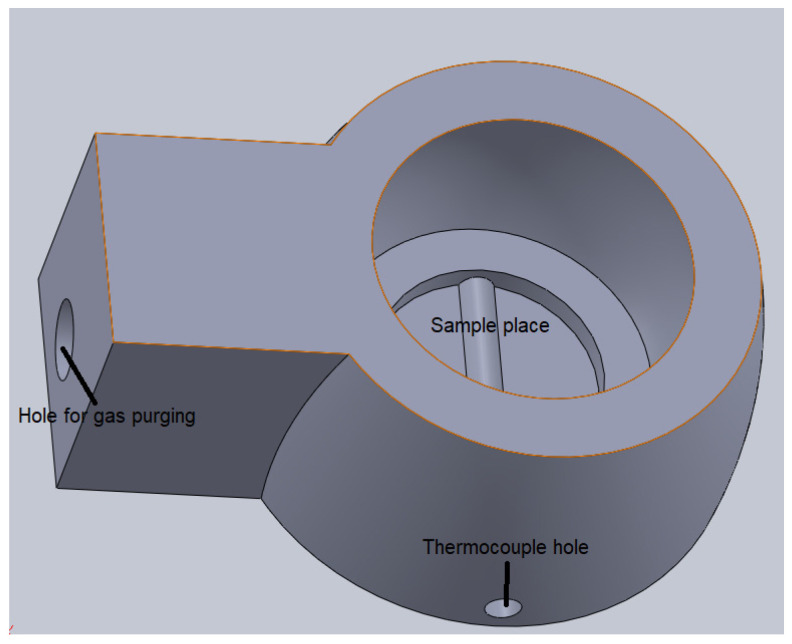
Ceramic crucible for sintering.

The sintering process was performed in a 3D-printed ceramic crucible (Materials Science Department from Transilvania University of Brasov), provided with a hole for gas purging and holes for fixing of thermocouple for measuring the temperature during sintering (Figure 2).

### 2.3. Materials Properties and Manufacturing Conditions

**Figure 3:**
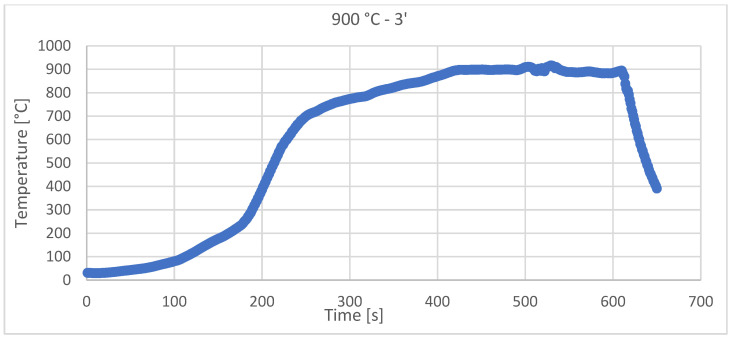
Solar sintering diagram at 900 °C, with 3 min holding time and with a 116.7 °C/min heating rate.

**Figure 4:**
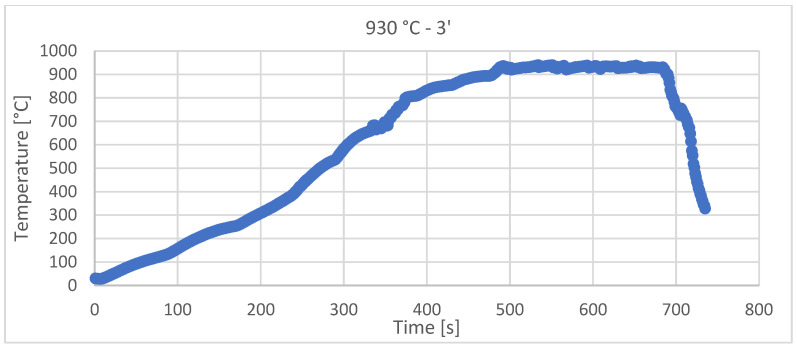
Solar sintering diagram at 930 °C, with 3 min holding time and with a 113.87 °C/min heating rate.

The theoretical heating rate was V_het_ =120 ± 10 °C/min, but the real heating rate for each sample (depending on solar flux incident on the sample) is presented in Table 1. The practical sintering diagrams are presented in Figure 3 and Figure 4 for each sintering temperature.

Cylindrical specimens with dimensions D × H = 16 mm × 30 mm were used and uniaxially pressed with a force of F = 90 kN in a metallic die.

Figure 3 and Figure 4 show the recordings made during the sintering with CSE of the samples, using Data Logger recorder—Pyro Tracer (model C.A. 650) and Type K thermocouples (maximum temperature—1300 °C).

Even though there were sometimes variations in the light intensity/wind that moved the reflecting mirrors, the temperature control with a variation of ±10 °C was achieved for all of the samples.

## 3. Results and Discussion

### 3.1. Microhardness

The microhardness was measured in nine points; the minimum and maximum values were eliminated; and from the remaining seven measurements, the arithmetic mean was calculated.

The study was conducted in the “filled” area—the area with thick walls—and in the “hollow” areas—that is, in the areas with thin copper walls resulting from the dissolution of the PVA model.

**Figure 5:**
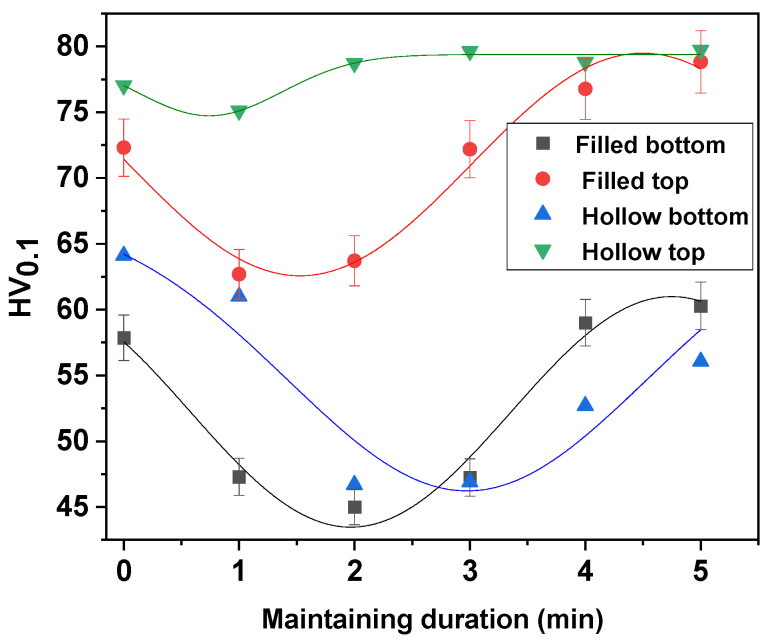
The microhardness obtained in the case of the sintering heat treatment at 900 °C.

**Figure 6:**
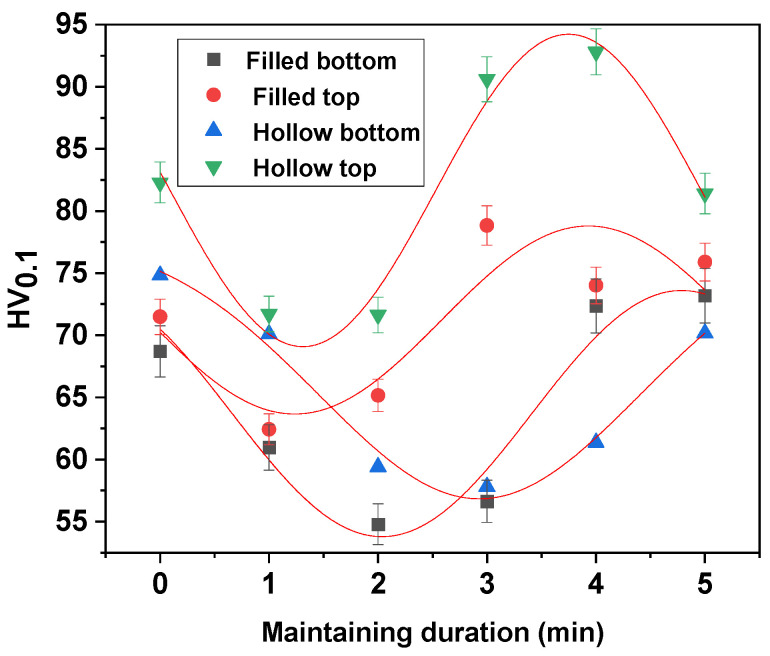
The microhardness obtained in the case of the sintering heat treatment at 930 °C.

Figure 5 and Figure 6 show the values of HV microhardness measured in the upper part of the sample (where the solar radiation was direct) and in the lower part (where the heat was transmitted through conductivity) in comparison with the solid and thin-walled samples.

Under normal conditions, copper as a metal presents a hardness of 50–110 HV [21,22,23]. When obtaining samples by pressing, a cold plastic deformation of the powder granules occurs, having the characteristic appearance of twins. Twinning is usually associated with internal tensions, and for this reason, the pressed samples have a higher hardness, with an average of 119.9 HV.

When samples are sintered, there is an ongoing relief in the internal stresses due to annealing [23].

Following the sintering process, the hardness of the samples presents different values as a function of different processes that occur in their obtaining:

- A uniform structure in the samples is realized through the disappearance of the boundaries between the metal granules.
- The remaining gaps from the stage when the samples are obtained by pressing participate in this process.
- In the case of all samples, a mechanical binding of the metal granules is noticed due to the pressing, which leads to an increase in the hardness, while in the heat-treated samples at 930 °C a bonding is produced due to the temperature.
- Even if the hardness variations are higher in the case of sintered samples at 930 °C, they are the highest.

### 3.2. Microscopic Analysis

The microscopic analysis was performed with a Nikon Eclipse MA 100 metallographic microscope (Nikon Corp., Tokyo, Japan). For the structure and morphology analysis, the next samples have been embedded into acrylic resin and leveled using an automatic metallographic Phoenix Beta polishing device from Buehler (with Al_2_O_3_ suspension and 0.05 μm grit).

#### 3.2.1. NS—Non-Sintered Sample

**Figure 7:**
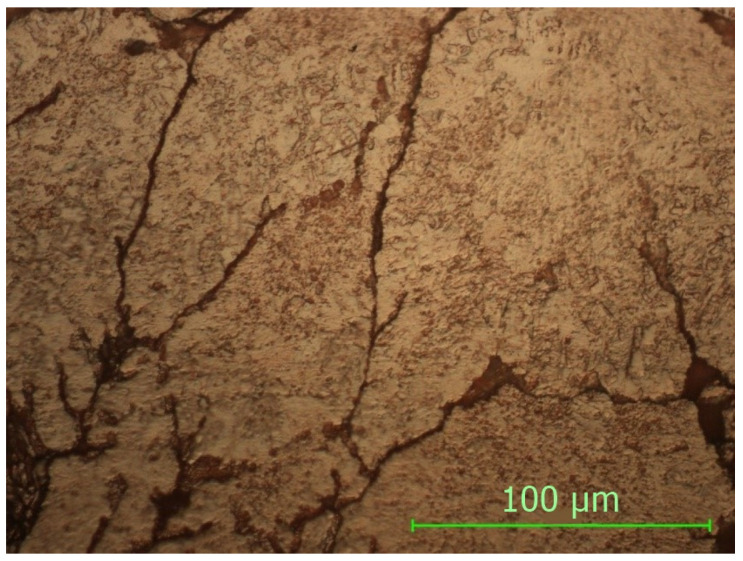
Non-sintered sample, magnification 500×.

From the analysis of the microstructure in Figure 7, a plastic deformation can be observed, which appeared at the moment during pressing in the mold. Due to the different internal energies resulting from the plastic deformations as well as the different reaction to the reagent, there are minor differences in the microstructure.

The spaces and the limits between the copper particles are small due to the pressing, with the mechanical binding appearing, which gives it a good mechanical resistance to the sample as well as a high microhardness.

#### 3.2.2. STW_900_

**Figure 8:**
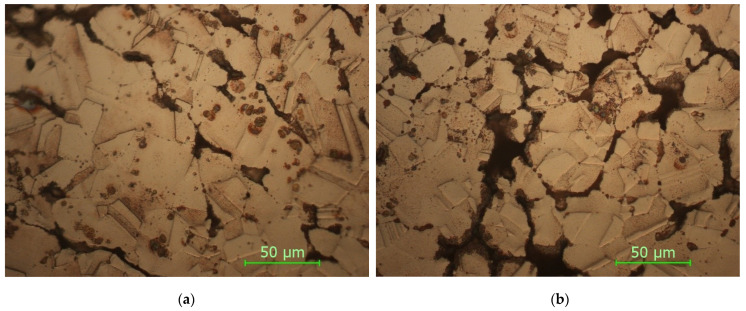
Sample STW900: (**a**) upper side; (**b**) bottom side (magnification 500×).

In Figure 8 are presented the microstructures in the case of the sample with thin walls sintered at 900 °C. 

#### 3.2.3. SFW900

**Figure 9:**
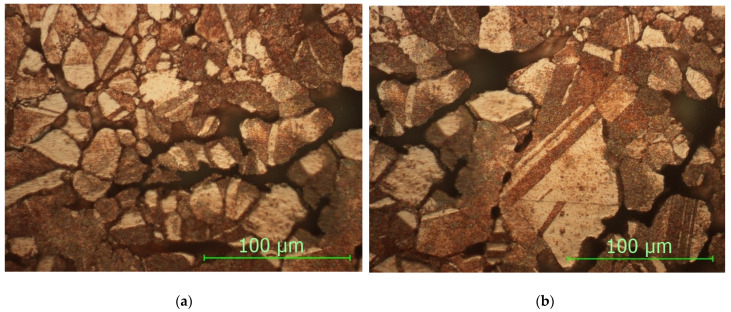
Sample SFW900: (**a**) upper side; (**b**) bottom side (magnification 500×).

In Figure 9 are presented the micrographs for the sample with fully walls sintered at 900 °C, with a holding time of 5 min.

As it can be seen from the micrographs presented in Figure 8 and Figure 9 and corroborating the values of HV microhardness presented in Figure 5, the following influencing factors could be found:

- The direct radiation facilitates the binding at certain temperatures and thus the sintering of the material takes place, resulting in a hardness close to the maximum hardness of Cu.
- In the case of samples with thin walls, the same amount of heat is transmitted in the mass of the part much more easily than in solid parts, with the results being decreases in the size, and number of defects and gaps and an increase in microhardness.
- Even if Cu is a good thermal conductor when full parts are concerned, the sintering process is affected, resulting in parts with a lower hardness.
- The variation in the microhardness is uniform and similar in the case of solid samples in the upper part and in the lower part.
- By applying the heating process, slightly larger gaps can be observed in the samples due to uneven expansion so that, locally, the effect of mechanical bonding is canceled and, for thermal bonding, the temperature/time of treatment is insufficient.

#### 3.2.4. STW930 

**Figure 10:**
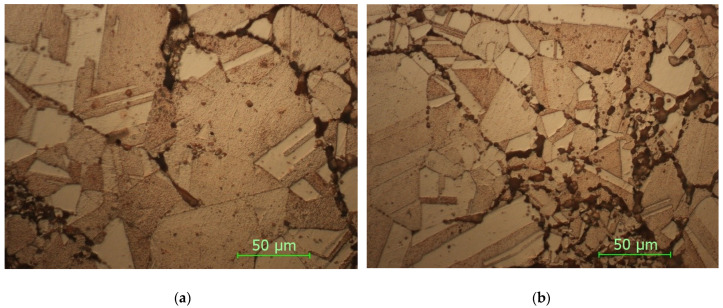
Sample SFW930: (**a**) upper side; (**b**) bottom side (magnification 500×).

In Figure 10 are presented the micrographs for the sample with thin walls sintered at 930 °C, with a holding time of 5 min.

#### 3.2.5. SFW930

**Figure 11:**
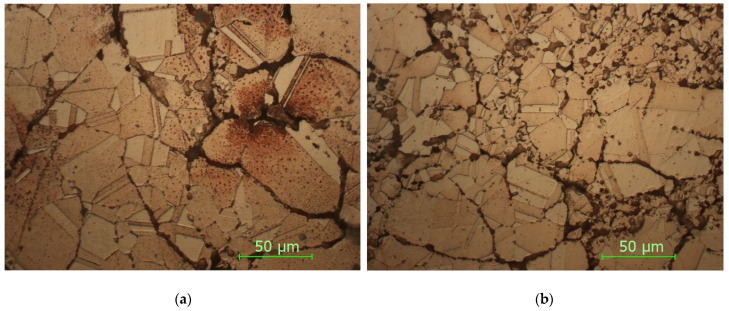
Sample SFW930: (**a**) upper side; (**b**) bottom side (magnification 500×).

In Figure 11 are presented the micrographs for the sample with fully walls sintered at 930 °C, with a holding time of 5 min.

As it can be seen from the micrographs presented in Figure 10 and Figure 11 and corroborating the values of microhardness presented in Figure 6, the following influencing factors were found to take place:

- Direct radiation facilitates the binding at temperature (as in the case of sintering at 900 °C), and thus, the material is sintered, resulting in a high hardness.
- Tt is possible to observe the disappearance of the boundaries between the grains, a phenomenon characteristic of the thermal treatments with maintenance at high temperatures.
- The results of the microhardness in the case of the heat treatment at 930 °C do not vary much, being close both to the solid/thin-walled samples and to the surface where direct radiation was employed and to the lower part where the heat propagated through thermal conductivity.
- Maintaining a higher temperature can cause an increase in the size of the crystals, resulting in polyhedral crystals with twin boundaries and orientation in bands.
- In the case of heat treatments performed at 930 °C, the clear influence of the increase in the temperature and in the holding time can be observed by decreasing the defects in the microstructures as well as by increasing the microhardness.
- By comparing the micrographs obtained at the sintered samples at 900 °C and 930 °C, a clear improvement in the microstructures can be seen by decreasing the number of defects and increasing the number of polyhedral crystals, which leads to an increase in the microhardness.

## 4. Conclusions

Solar sintered parts with controlled internal geometry were obtained in this study via the negative part method, achieved through 3D printing and powder metallurgy. This approach has not been used until now, and it can be considered an innovation in the field. The proposed configuration of the copper parts is difficult to achieve using other technologies.

- Generally, due to higher production costs, professional 3D printing technologies are not suitable for mass production. Instead, due to the short time frame from designing to obtaining the physical product, they are more suitable for prototypes and it is the best solution for checking/validating the changes made to parts before their mass production. In this way, small series or prototypes can be obtained.
- With an increase in the temperature from 900 °C to 930 °C, the particles practically bind better and the gaps (pores) disappear.
- The contours of the grains are noticeable at 900 °C, even if the adhesion of the particles is more obvious at 930 °C, a fact confirmed by the microhardness values.
- In the case of heat treatment at 930 °C, a stronger finishing of the structure was obtained.
- By comparison, the solid sample/the sample with gaps at the same temperature and holding time shows a reduction in the gaps (porosities), a good compaction and embedding of the particles clearly superior to the samples with thin walls.
- Internal microporosity is random due to the technological process specific to powder metallurgy and is dictated by the distribution of granules upon pressing.
- Using 3D printing technology with the powder processing technology and solar sintering shows combined benefits.
- The 5 min holding time for these samples at 930 °C proved to be the best after we analyzed and compared all of the microstructures made and the values of the microhardness. Additionally, 5 min was proven to be the average holding time based on the experiments.
- Using solar energy in the sintering process is feasible because of its advantages: clean energy, inexhaustible energy, environmentally friendly, and shorter heat treatment durations.
- The results presented in this paper have determined that concentrated solar energy sintering can be successfully applied to sinter copper heat exchanger parts with a controlled 3D-printed morphology.

## Figures and Tables

**Table 1:** The sintering parameters.

No. Crt.	Temperature (°C)	Holding Time (min)	Theoretical Heating Rate (°C/min)	Practical Heating Rate (°C/min)
1	900	0	120 ± 10	112.24
2		1		125.6
3		2		126.3
4		3		116.7
5		4		122.6
6		5		110.8
7	930	0	120 ± 10	111.7
8		1		115.4
9		2		119.9
10		3		113.87
11		4		118.2
12		5		117.1

## Data Availability

Not applicable.
